# Effect of the Articular Surface Incongruency on Surgical Outcome of the Distal Radius Fracture

**DOI:** 10.1155/2022/8357675

**Published:** 2022-03-09

**Authors:** Chang-Hun Lee, Youngmin Kwon, Il Youn Jung, Bong-Gun Lee, Sung Jae Kim

**Affiliations:** ^1^Department of Orthopaedic Surgery, Hanyang University College of Medicine, Seoul, Republic of Korea; ^2^Department of Orthopaedic Surgery, Dongtan Sacred Hospital, Hallym University, Hwaseong, Republic of Korea

## Abstract

**Purpose:**

The aim of this study was to demonstrate the joint fragment that mostly affects the outcome of the distal radius fracture surgically treated with a volar locking plate (VLP).

**Methods:**

The outcomes of 69 patients with the distal radius fractures were evaluated at their final follow-up. The articular surface was divided into six specific fragments, and computed tomography (CT) was used to evaluate the degree of mismatch of each fragments. A plain radiograph was also obtained for evaluation of the distal radius alignment. Clinical outcomes were measured by using the Disabilities of the Arm, Shoulder and Hand (DASH) and Modified Mayo Wrist Score (MMWS). Univariate analyses were performed, with subsequent multiple logistic regression analyses.

**Results:**

The mean follow-up period was 14.8 (range, 12 to 52) months. The group with a worse DASH score showed significantly greater mismatch in the volar and dorsal lunate facets, as well as the central depression of the distal radius (*p* = 0.042, 0.031, and 0.023, respectively). There was a significant positive correlation between the DASH score and degree of mismatch of the dorsal lunate facet and central depression of the distal radius (*p* = 0.040 and 0.011, respectively). Groups with worse MMWS showed significantly greater mismatch in the dorsal lunate facet (*p* = 0.025). There was a significant negative correlation between MMWS and abnormal ulnar variance and mismatch of the dorsal lunate facet and central depression of the distal radius (*p* = 0.041, 0.004, and 0.018, respectively). The result of multiple logistic regression analysis demonstrated that a mismatch of the dorsal lunate facet is a significant predictor for a worse MMWS (odds ratio = 3.072, *p* = 0.043).

**Conclusions:**

Articular surface mismatch of the dorsal lunate facet appears to mostly affect the surgical outcomes of the distal radius fractures using VLP. In cases where the dorsal lunate facet is heavily involved, surgeons should be cautious about its reduction and fixation.

## 1. Introduction

The distal radius is one of the most common fracture sites of the human skeleton [1]. Surgical management is currently favoured for displaced intra-articular fractures, as accura reduction and stable fixation are considered the key for a favourable long-term clinical outcome [2, 3]. Anatomical volar locking plates (VLPs) are widely used in the surgical management of the distal radius fractures. Recently, fragment-specific fixation devices were introduced, and their biomechanical properties and advantages in management of the complex intra-articular fractures have been reported [3–5].

Several studies have investigated the effect of specific articular fragment displacements of the distal radius on surgical outcomes [6–8]. These studies however relied on plain radiographs to assess articular surface congruity. Previous studies have demonstrated the superiority of computed tomography (CT) over plain radiography in assessment of articular surface congruity in the distal radius fractures [9, 10]. Additionally, previous studies about the effect of fragment-specific reduction status on the surgical outcome did not control for the confounding effect of injuries involving multiple articular fragments.

We evaluated reduction of each specific articular fragment by using postoperative CT scans. The purpose of this study was to determine which joint fragment mostly affects the outcome of the distal radius fractures repaired with VLP.

## 2. Patients and Methods

This prospective study was approved by the institutional review board of our institute. We reviewed the medical records of 123 consecutive patients who were surgically treated for an unstable fracture of the distal radius with a VLP at our institute between May 2012 and February 2014. The inclusion criteria were as follows: unstable fracture of the distal radius, surgical treatment with a VLP, performed implant removal surgery after fracture union, and availability of CT scans both preoperatively and at the final follow-up. Implant removal surgery after fracture union was indicated for patients who preferred removal of the implant and patients at risk of the flexor tendon irritation. The final follow-up CT scans were obtained one day following implant removal surgery. Patients were excluded if the fracture was extra-articular type and if there was a history of ipsilateral hand and wrist injury or an accompanying metaphyseal or diaphyseal ulnar fracture. Of the 123 patients, 29 patients had extra-articular distal radius fracture, and 11 patients were excluded because they did not undergo implant removal surgery; seven refused to participate in the study, two had a history of ipsilateral hand and wrist injury, three had an accompanying metaphyseal or diaphyseal ulnar fracture, and two were lost to follow-up. In total, 69 patients were included in the final study.

Distal radius fractures were internally fixated with a VLP (Synthes, Paoli, USA, or Acumed, Oregon, USA) in all patients. All surgeries included in this study were performed by a single, senior author. A postoperative splint was applied, which was changed to a wrist brace two weeks postsurgery. A range of finger motion exercises were permitted on the day of surgery. Wrist exercises commenced two weeks following surgery and were increased as tolerated.

### 2.1. Assessment of Articular Congruity

**Figure 1:**
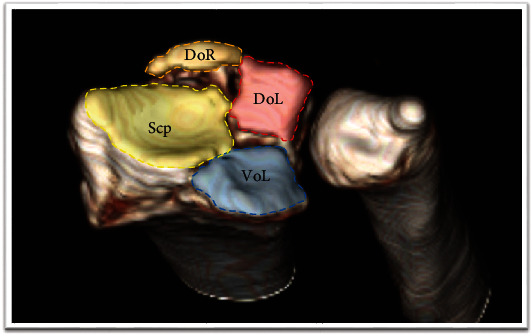
Initial CT scans. The articular surface was divided into six fragments: scaphoid facet (Scp), dorsal lunate facet (DoL), volar lunate facet (VoL), dorsal rim fragment (DoR), volar rim fragment, and central depression type. In this subject, the dorsal and volar lunate facet and dorsal rim fragment were involved.

**Figure 2:**
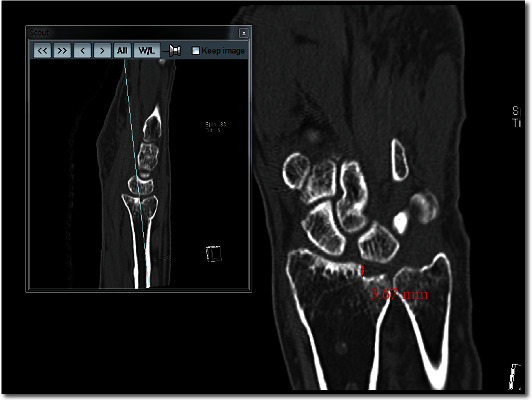
Final CT scans. Degrees of the articular gap of step-off of each involved fragment were measured.

In order to assess the reduction of each specific fragment of the distal radius, the articular surface was divided into six specific fragments: scaphoid facet, volar lunate facet, dorsal lunate facet, central depression, dorsal rim, and volar rim. Dorsal or volar fragments that contain less than 2 mm of the joint surface were defined as a rim fragment [7]. Articular surface mismatch was defined as any step-off or gap observed on CT. Assessment of articular surface mismatch was performed following the standard procedure. First, we examined the three-dimensional (3D) reconstructed articular surface image from preoperative CT scans to define the affected fragments (Figure 1). Then, on the final CT scans, the remaining step-off of the involved articular surface fragments was measured using PiViewSTAR measurement tools (PiView 5.08; Infinitt, Seoul, Korea) (Figure 2). The step-off of the scaphoid facet, both lunate facets, central portion, and volar and dorsal rim was measured in the coronal and sagittal reconstructed images. The step-off of the sigmoid notch was measured in the axial image of CT scans. The articular surface mismatch of each specific fragment was evaluated on a three-grade scale: “not involved,” “mismatch less than 2 mm,” and “mismatch more than 2 mm.” [11] Plain postero-anterior (PA) and lateral radiographs of the wrist were taken at the final consultation to evaluate the alignment of the distal radius. The PA radiograph allowed measurement of the radial length, radial inclination, and ulnar variance of the wrist joint. The volar tilt angle was measured using the lateral view. These measurements were recorded as either “within normal range” or “abnormal.” The normal ranges of each alignment parameter examined on simple radiographs were as follows: radial height, 8 to 18 mm; radial inclination, 13 to 30 degrees; ulnar variance, -4 to 2 mm; and volar tilt angle, 0 to 28 degrees [12].

To assess measurement reliability, all measurements were performed by a single observer at two times, followed by a second independent observer who was unaware of the initial measurements.

### 2.2. Clinical Outcome Assessment

All subjects completed the DASH and MMWS questionnaires at the day before implant removal surgery. The DASH instrument is a patient-reported scoring system developed by the Institute for Work and Health in Ontario and the American Academy of Orthopaedic Surgeons in 1994 [13]. It is comprised of 30 items that evaluate the symptoms and physical function of the arm, shoulder, and wrist. Each item is scored on a five-point ordinal scale. The total score is modified so that 100 points indicate maximum disability and 0 point indicates a normal wrist. The MMWS is a physician-reported scoring system. It is comprised of four 25-point sections; an evaluator's assessment of pain, active flexion/extension arc of the wrist joint, grip strength, and the ability to return to regular activities. Pain was assessed by the evaluator based on the patient's subjective description [14, 15]. The MMWS' score ranges from 0 to 100 points with higher scores indicating better result.

### 2.3. Statistical Analysis

To assess measurement reliability, we calculated Cohen's kappa value between repeated measurements done by a single observer and between measurements done by two independent observers. All the subjects were divided into two groups according to their final MMWS and DASH score, and two groups were compared. We set the DASH score of 29 points as the cut-off score for separating two groups. A recent review of the DASH questionnaire described that the score ranging from 0 to 29 could be considered “no longer considering their upper limb disorder a problem.” [16]

For MMWS scores, an excellent result is defined as 90 to 100, good is 80 to 89, fair is 65 to 79, and poor is less than 65 [15]. We set the cut-off score for MMWS as 80 points for separating two groups, the lower limit of good functional result.

Spearman's rho and Pearson's correlation coefficients were calculated to analyse the correlation between several anatomical parameters and clinical outcome.

Parameters with *p* < 0.05 in the univariate analyses were selected as independent variables for multiple logistic regression analysis. Multiple logistic regression analyses were performed to evaluate the cause-effect relationship between the surgical outcome and mismatch of each specific fragment and to control for the confounding effect of each parameter.

## 3. Results

**Figure 3:**
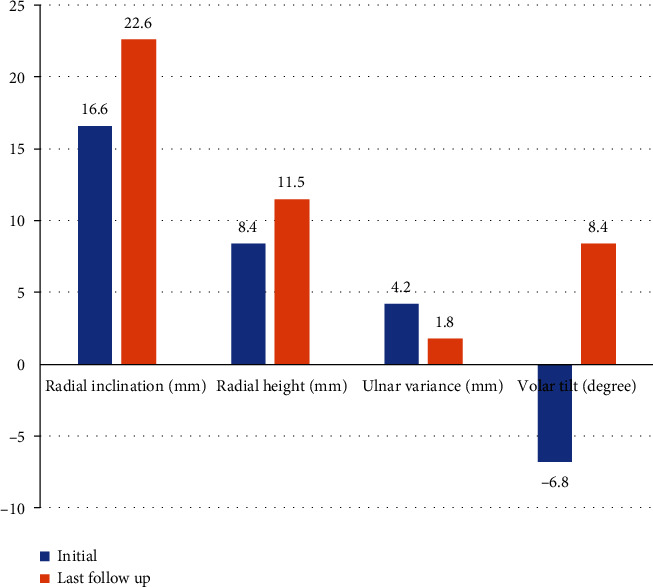
Mean of radiologic alignment parameters between the initial and last follow-up.

**Figure 4:**
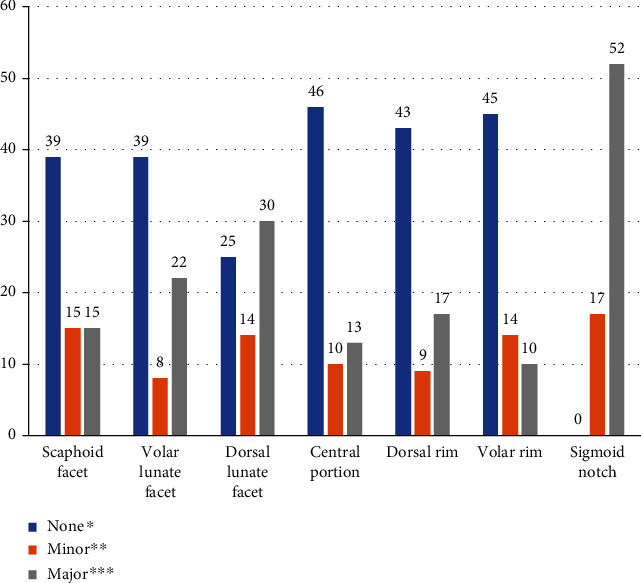
Number of patients between non-step-off and step-off depending on the location of the fracture.

No patients showed specific complications after surgical treatment. Baseline patient demographic data are listed in Table 1. The mean age of patients was 54.8 (range, 25–73) years. The mean follow-up duration was 14.8 (12 to 52) months. Cohen's kappa values of all the measurements in this study were between 0.713 and 0.835. Radiologic results of variable parameters between the initial and last follow-up are depicted in Figure 3. The results of step-off within each facet observed at the last follow-up are depicted in Figure 4. Comparisons of outcomes between the two groups divided by the final DASH score are listed in Table 2. There were significantly more incidences of AO type C fracture in the group with the worse final DASH score (*p* = 0.029). The degree of mismatches of the volar and dorsal lunate facets and the central depression were significantly greater in the group with the worse final DASH score (mean rank, 32.9 vs. 41.7, *p* = 0.042; mean rank, 32.4 vs. 43.6, *p* = 0.031; and mean rank, 33.2 vs. 41.1, *p* = 0.023, respectively).  Table 3 lists the correlation between the DASH score and several variables. The degree of mismatch of the dorsal lunate facet and the central depression showed a significant positive correlation with the final DASH scores.

Comparisons of outcomes between the two groups divided by the final MMWS are listed in Table 4. The degree of mismatch of the dorsal lunate facet was significantly greater in the group with the worse final MMWS score (mean rank, 29.5 vs. 39.5, *p* = 0.025).  Table 5 lists the correlation between MMWS and several variables. Ulnar variance, the degree of mismatch of the dorsal lunate facet, and the central depression showed a significant negative correlation with the final MMWS.

Multiple logistic regression analysis for the final DASH score > 29 group (worse DASH score) demonstrated that the degree of mismatch of the dorsal lunate facet had a tendency to be predictive for a worse DASH score; however, this was not statistically significant (*p* = 0.062). The degree of mismatch of the dorsal lunate facet was predictive for a lower MMWS (odds ratio = 3.072, *p* value = 0.043), despite controlling for several confounding factors (Table 6).

## 4. Discussion

To the best of our knowledge, this is the first investigation to use postoperative CT scans to evaluate the effects of each articular surface fragment on surgical outcomes of the distal radius fracture. Our results demonstrate that articular surface mismatch of the dorsal lunate facet seems to be predictive for a worse MMWS score.

Previous studies into the treatment outcome of distal radius fractures have drawn a number of conclusions, which are important to consider when evaluating our results. Not a few previous studies have noted that the functional outcome of the wrist as perceived by the patient does not necessarily correlate with the degree of objective compromise of the joint. Finsen et al. reviewed 260 patients with distal radius fractures that had been treated conservatively [17]. Their results indicated that the final alignment of the distal radius had little effect on the functional outcome. They described that almost all degrees of malunion still resulted in good function. Furthermore, poor functional results may be seen in cases with normal alignment [17]. Goldfarb et al. followed 16 young patients with intra-articular distal radius fractures who were surgically treated with open reduction and internal fixation over an average 15-year follow-up period. They noted that radiocarpal arthrosis seemed to worsen over time with such fractures. They also noted, however, that despite advanced arthrosis in the radiocarpal joint, patients had a high functional level at long-term follow-up [18]. Our study also showed that the mismatch of the scaphoid facet and sigmoid notch was not significantly correlated with the functional outcomes.

However, it seems important to consider the ceiling effect of each outcome measuring score system when comparing the clinical outcome of distal radius fractures [19]. There may still be a difference between the clinical outcomes of an accurately reduced distal radius fracture and a malunited one. Gruber et al. noted that subsequent arthrosis of the wrist joint caused by intra-articular distal radius fractures seemed to negatively impact patients' subjective well-being and quality of life despite not affecting the objective outcomes [20]. Furthermore, there are a number of studies describing the importance of certain anatomical factors related to functional outcome in distal radius fractures. The volar tilt angle, for example, is ascribed great importance for the effective functioning of the wrist. It influences the degree of wrist flexion and the hand's grip strength and causes symptomatic incongruity of the distal radioulnar joint (DRUJ) [21–24]. Anzarut et al. also described the volar tilt angle as the most important radiological parameter for determining the alignment of the distal radius [23].

A small number of previous studies have investigated the relation between specific articular fracture fragments of the distal radius fractures and their surgical outcomes. Kim and Cho described surgical outcomes of distal radius fractures with displaced dorsal rim fragments [7]. They compared two groups of 23 distal radius fracture cases. They concluded that there was no statistically significant difference between the surgical outcomes of distal radius fractures with and without dorsal rim fragment displacement. Marcano et al. investigated surgical outcomes of the distal radius fractures with volar rim fragment involvement [8]. They described similar surgical outcomes for distal radius fractures of this type compared with those that involved other types of intra-articular fragments. These results are in accordance with our findings. We found that mismatches of volar and dorsal rim fragments showed no significant result in any of the statistical analyses we conducted.

Our data demonstrates that the volar and dorsal lunate facets are most frequently involved in the distal radius fractures, and mismatches of the articular surface also remained most frequently at these facets after surgery. There may be two explanations for this. First, the standard volar surgical approach using intervals between the flexor carpi radialis tendon and the radial artery may not be sufficient to accurately reduce the volar and dorsal lunate facets. Second, in some cases, the conventional VLP system seems to be insufficient to support the axial force that arises at the radiolunate articulation. This assumption is in accordance with the findings of Harness et al. [6] They reviewed seven patients with volar shearing distal radius fractures who lost fixation of a volar lunate facet fragment and had carpal displacement following open surgical treatment [6]. All cases were initially considered to have been adequately reduced and fixated; however, four patients required repeat open surgical fixation, and one underwent a wrist arthrodesis. They noted that the anatomy of this region may have prevented standard surgical fixation devices from supporting the entire volar surface effectively [6].

The main advantage of this study is that it was able to use CT to examine the reduced articular surface in more detail. This allowed us to use statistics to control for the confounding effect of accompanying different articular fragments. Therefore, we believe that this study provides current and reliable clinical information on the relationship between specific joint fragments of the distal radius fractures and their surgical outcomes. We have revealed that dorsal lunate facet fragments have a significant cause-effect relationship with the surgical outcome. When using the VLP system in the surgical treatment of intra-articular distal radius fractures with large dorsal lunate facet displacement, surgeons should be cautious about accurate reduction and rigid fixation of this fragment.

There are some limitations to this study. First, we were only able to evaluate the result of a distal radius fracture in the short term. It should be noted, however, that previous studies did not show a great difference in outcomes between the first and second year of follow-up [20, 25]. Second, we did not evaluate symptoms specific to the distal radioulnar joint (DRUJ). Evaluation of signs and symptoms specific to DRUJ may have helped to explain the underlying pathophysiology of our results. Further studies with longer follow-up periods and more symptom-specific analyses may be able to provide more information.

## Figures and Tables

**Table 1:** Baseline data of patients.

Number	69
Age (years, range, SD)	54.8 (25 to 73, 9.94)
Sex (male, %)	18 (26.1%)
Side (dominant side, %)	30 (43.5%)
Delay to surgery (days, range, SD)	3.9 (0 to 15, 2.98)
Follow-up duration (months, range, SD)	14.8 (12 to 52, 7.71)
Fracture classification (AO)	
*B*	14 (20.3%)
*C*	55 (79.7%)

SD: standard deviation.

**Table 2:** Relation between the result of the patient-reported rating scale (DASH) and patients' variables.

	Group with DASH ≤29 points (*n* = 53)	Group with DASH >29 points (*n* = 16)	*p* value
Age at injury	54.9 (9.89)	54.5 (10.39)	0.955
Sex (male : female)	11 : 42	7 : 9	0.102
Side (% of dominant side)	25 (47.2%)	5 (31.3%)	0.389
Follow-up duration (months)	15.4 (8.63)	13.0 (2.60)	0.275
Delay to surgery (days)	4.1 (3.18)	3.4 (2.19)	0.551
Fracture type (AO) (*B* : *C*)	14 : 39	0 : 16	0.029
Initial injury of the ulnar styloid process (none : tip : base)	17 : 6 : 30	4 : 5 : 7	0.705
Radial inclination (% of normal)	40 (75.5%)	11 (68.8%)	0.746
Radial height (% of normal)	41 (77.4%)	13 (81.3%)	1.0
Ulnar variance (% of normal)	41 (77.4%)	11 (68.8%)	0.518
Volar tilt (% of normal)	37 (69.8%)	10 (62.5%)	0.760
Mismatch of the scaphoid facet^∗^	33.0	41.5	0.058
Mismatch of the volar lunate facet^∗^	32.9	41.7	0.042
Mismatch of the dorsal lunate facet^∗^	32.4	43.6	0.031
Mismatch of the central portion^∗^	33.2	41.1	0.023
Mismatch of the dorsal rim^∗^	36.0	31.7	0.240
Mismatch of the volar rim^∗^	35.3	34.1	0.738
Mismatch of the sigmoid notch^∗^	36.8	33.7	0.583

^∗^Results were presented with the mean rank.

**Table 3:** Correlation between DASH and several variables.

	Spearman's rho	*p* value
Age	-0.012	0.923
Delay to surgery^∗^	-0.057	0.643
Follow-up duration^∗^	-0.152	0.213
Fracture type	0.109	0.374
Initial injury of the ulnar styloid process	-0.130	0.289
Radial inclination at final	-0.130	0.286
Radial height at final	-0.015	0.903
Ulnar variance at final	0.128	0.293
Volar tilt at final	0.002	0.990
Mismatch of the scaphoid facet	0.153	0.208
Mismatch of the volar lunate facet	0.136	0.264
Mismatch of the dorsal lunate facet	0.248	0.040
Mismatch of the central portion	0.304	0.011
Mismatch of the dorsal rim	-0.109	0.375
Mismatch of the volar rim Mismatch of the sigmoid notch	0.017 -0.123	0.891 0.308

^∗^Pearson's correlation coefficient.

**Table 4:** Relation between the result of the physician-reported rating scale (MMWS) and patients' variables.

	Group with MMWS ≥ 80 points (*n* = 31)	Group with MMWS < 80 points (*n* = 38)	*p* value
Age at injury (SD)	53.8 (11.54)	55.7 (8.48)	0.663
Sex (male : female)	7 : 24	11 : 27	0.593
Side (% of dominant side)	16 (51.6%)	14 (36.8%)	0.373
Follow-up duration (months, SD)	16.0 (8.86)	13.8 (6.59)	0.136
Delay to surgery (days)	4.4 (3.40)	3.63 (2.59)	0.348
Fracture type (AO) (*B*: *C*)	3 : 23	6 : 32	0.373
Initial injury of the ulnar styloid process (none : tip : base)	7 : 5 : 19	14 : 6 : 18	0.485
Radial inclination (% of normal)	21 (67.7%)	30 (78.9%)	0.409
Radial height (% of normal)	23 (74.2%)	31 (81.6%)	0.561
Ulnar variance (% of normal)	25 (80.6%)	27 (71.1%)	0.263
Volar tilt (% of normal)	23 (74.2%)	24 (63.2%)	0.437
Mismatch of the scaphoid facet^∗^	34.1	35.8	0.661
Mismatch of the volar lunate facet^∗^	33.8	36.0	0.552
Mismatch of the dorsal lunate facet^∗^	29.5	39.5	0.025
Mismatch of the central portion^∗^	32.1	37.3	0.079
Mismatch of the dorsal rim^∗^	36.4	33.9	0.420
Mismatch of the volar rim^∗^	32.7	36.9	0.172
Mismatch of the sigmoid notch	37.3	34.1	0.520

^∗^Results were presented with the mean rank.

**Table 5:** Correlation between MMWS and several variables.

	Spearman's rho	*p* value
Age	0.052	0.674
Delay to surgery^∗^	0.107	0.382
Follow-up duration^∗^	0.327	0.006
Fracture type	-0.212	0.081
Initial injury of the ulnar styloid process	0.100	0.415
Radial inclination at final	0.122	0.318
Radial height at final	0.075	0.542
Ulnar variance at final	-0.247	0.041
Volar tilt at final	-0.123	0.315
Mismatch of the scaphoid facet	-0.196	0.107
Mismatch of the volar lunate facet	-0.094	0.443
Mismatch of the dorsal lunate facet	-0.345	0.004
Mismatch of the central portion	-0.284	0.018
Mismatch of the dorsal rim	0.135	0.267
Mismatch of the volar rim	-0.155	0.203
Mismatch of the sigmoid notch	0.074	0.537

^∗^Pearson's correlation coefficient.

**Table 6:** Result of the multiple logistic regression model between the worse result of MMWS and articular surface mismatch.

	*p* value	Odds ratio	95% CI of odds ratio
Age	0.464	1.020	0.968 to 1.074
Follow-up duration	0.305	0.958	0.884 to 1.039
Fracture type	0.634	1.398	0.351 to 5.567
Ulnar variance	0.726	1.266	0.339 to 4.731
Mismatch of the dorsal lunate facet	0.043	3.072	1.035 to 9.121
Mismatch of the central portion	0.269	2.802	0.451 to 17.397

CI: confidence interval.

## Data Availability

Raw data of the current study can be provided upon request.
